# Necroptosis in vascular cognitive impairment: mechanisms and therapeutic potential

**DOI:** 10.3389/fnagi.2025.1599773

**Published:** 2025-06-25

**Authors:** Shufei Wei, Lin Cheng, Chunxiao Shen, Zefen Li, Jiahui Teng, Liangliang Wang, Xiaorong Zhang

**Affiliations:** ^1^Department of Pathology, Affiliated Hospital of Jiujiang University, Jiujiang, Jiangxi, China; ^2^Department of Pathology, Jiujiang Clinical Precision Medicine Research Center, Jiujiang, Jiangxi, China; ^3^Department of Neurology, Affiliated Hospital of Jiujiang University, Jiujiang, Jiangxi, China

**Keywords:** necroptosis, chronic cerebral hypoperfusion, vascular cognitive impairment, cerebral ischemia, neurodegenerative disease

## Abstract

Cerebral ischemia and hypoxia play key roles in the occurrence and development of vascular cognitive impairment (VCI). However, the pathophysiology of VCI remains unclear. Necroptosis is a non-cysteine-dependent form of cell death mediated by serine/threonine kinases receptor-interacting protein kinase-1 and -3 and mixed lineage kinase domain-like protein. A search of PubMed and Web of Science was conducted using terms related to VCI and necroptosis. Necroptosis is important in neuroinflammation, neuronal loss, blood–brain barrier dysfunction, and demyelination. Cerebral ischemia activates the necroptotic pathway, and necroptosis inhibitors have a significant inhibitory effect on brain injury. This review focuses on the pathogenesis of VCI and clarifies the core regulatory mechanism of necroptosis in vascular dementia, which lays a scientific foundation for cognitive impairment prevention and treatment by targeting necroptosis in VCI.

## Graphical abstract

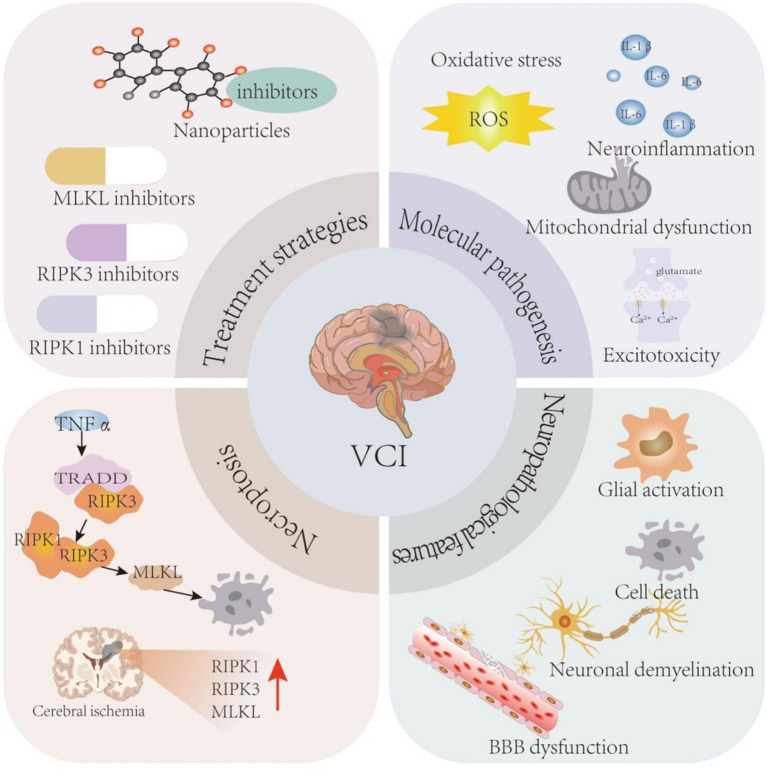

## 1 Introduction

Research on vascular cognitive impairment (VCI) is mainly focused on the influence of vascular factors on any degree of cognitive function, from mild deficits to prodromal and fully developed dementia (Badji et al., 2023; Chang Wong and Chang Chui, 2022). In 2018, 50 million people are estimated to be affected globally, of which approximately 25% are Chinese (Lu et al., 2020), and this number is expected to triple by 2050 (Liu et al., 2024; Guo et al., 2023). Moreover, patients with VCI have a high mortality rate (Ren and Qu, 2023).

Chronic cerebral hypoperfusion (CCH) and cerebral hypoxia are the key causes of VCI (Rajeev et al., 2023). The molecular and cellular pathogenic mechanisms of VCI are only partially elucidated. Recent studies have shown that necroptosis plays an important role in brain tissue injury caused by cerebral ischemia and hypoxia (Nikseresht et al., 2019). However, despite growing evidence on necroptosis in ischemic brain injury, its precise role in VCI pathology remains unclear.

**Figure 1:**
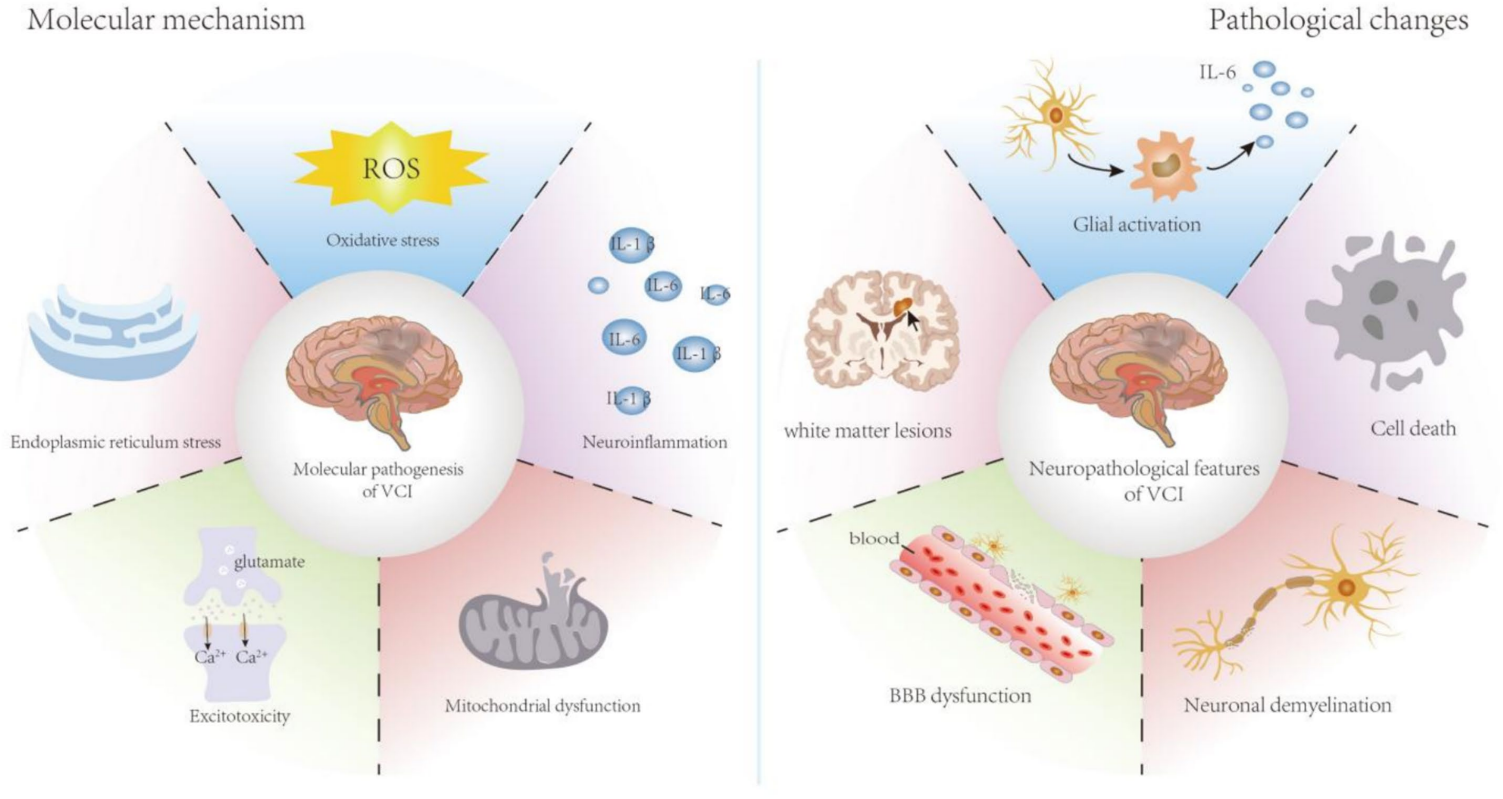
Molecular mechanism and pathological changes of VCI. The molecular mechanisms of VCI mainly include oxidative stress, neuroinflammation, and mitochondrial dysfunction. The pathological changes of VCI mainly include glial activation, cell death, BBB dysfunction, neuronal demyelination, and white matter lesions. BBB, blood brain barrier; IL, interleukin; ROS, reactive oxygen species; VCI, vascular cognitive impairment.

In this review, we summarize the current knowledge of the molecular mechanisms and pathological changes in VCI, describe the role of necroptosis in the pathological changes caused by cerebral ischemia and hypoxia, and discuss the application of necroptosis inhibitors as potential therapeutic interventions for VCI (Figure 1).

## 2 Research status of the pathogenesis of VCI

The concept of VCI was first proposed by Hachinski and Bowler (1993). This concept has gradually replaced vascular dementia to describe cognitive decline caused by cerebrovascular diseases (Hachinski and Bowler, 1993; van der Flier et al., 2018). VCI can be divided into mild and severe forms; the latter includes post-stroke, subcortical ischemic vascular, multi-infarct, and mixed dementias (Skrobot et al., 2018; Masserini et al., 2023). Several mechanisms play a role in VCI progression; however, pathological mechanisms involve reduced cerebral blood flow, neuronal death, glial cell activation, white matter lesions (WMLs), blood–brain barrier (BBB) damage, and endothelial dysfunction. Oxidative stress and inflammation are the two main underlying mechanisms (Kalaria et al., 2024; Fitzgibbon-Collins et al., 2021). However, a thorough understanding of this complex disease is lacking. Therefore, a consensus on the underlying causes of VCI is needed.

Animal models used in VCI research typically mimic cerebral ischemia, including CCH, to simulate the underlying pathology of VCI. The whole brain is placed in a state of ischemia and hypoxia, resulting in progressive and persistent brain damage, such as energy metabolism imbalance, neuroinflammation, and white matter demyelination (He et al., 2023; Zheng et al., 2023). Cerebral blood flow reduction is also observed in VCI patients, causing neural and vascular changes such as the activation of glial cells, BBB dysfunction, demyelination, and endothelial cell (EC) damage (Zhang et al., 2023; Cheng et al., 2024). Cerebral ischemia is linked to known mechanisms of VCI, including neuroinflammation, oxidative stress, neuronal death, and energy imbalance (Rajeev et al., 2023; Zhao et al., 2023). In the following section, we review the pathogenesis of VCI caused by cerebral ischemia in detail.

### 2.1 Molecular pathogenesis of VCI

#### 2.1.1 Oxidative stress

Oxidative stress results in accumulation of numerous oxidative intermediates, such as reactive oxygen species (ROS) and reactive nitrogen radicals, leading to tissue damage and cell death (Martemucci et al., 2023; Maiese, 2023). Cerebral ischemia and hypoxia in VCI increases ROS levels in cerebrovascular ECs (Zeylan et al., 2024) and can cause disruption of Ca^2+^ homeostasis in the brain, impairing the function of the mitochondrial electron transport chain (ETC) in ECs. Activation of ROS enzymes, such as nicotinamide adenine dinucleotide phosphate oxidase (Nox), xanthine oxidase, and nitric oxide synthase (NOS), causes the production of excess ROS to further promote the occurrence of oxidative stress (Wang et al., 2024; Wang et al., 2023). In rodent models of CCH, Nox2 levels in the brain and in hippocampal neurons are increased and associated with cognitive impairment (Alfieri et al., 2022; Choi et al., 2014). Oxygen generates other ROS, such as hydrogen peroxide (H_2_O_2_) and hydroxyl free radicals, through a series of disproportionation reactions. *In vitro* experiments using cells exposed to H_2_O_2_ to simulate an oxidative stress injury have been performed (Song et al., 2020; Wang et al., 2021). Antioxidant enzymes such as superoxide dismutase, glutathione peroxidase, and catalase are decreased in the peripheral blood of VCI patients, whereas the oxidative stress markers malondialdehyde (MDA), reactive ROS, and lactate dehydrogenase are increased (Krishnan and Rani, 2014; Xue et al., 2017). Oxidative stress is the basic pathological process of VCI; however, the mechanism has not been fully elucidated, and further in-depth study is needed.

#### 2.1.2 Neuroinflammation

Neuroinflammation is an immune cascade mediated by microglia and astrocytes in the central nervous system (CNS) (Sanz et al., 2024; Villa-Gonzalez et al., 2024), which is caused by pathological injuries such as infection, ischemia, and hypoxia (Titisari et al., 2024; Thiankhaw et al., 2024). Neuroinflammation triggered by cerebral ischemia and hypoxia in a VCI mouse model causes WMLs, cognitive dysfunction, and learning and memory deficits. In addition, various proinflammatory factors, such as interleukin beta 1 (IL-1β) and IL-6, are significantly increased in brain tissue and serum (Gao et al., 2024; Tang et al., 2024). Neuroinflammation induces neuronal dysfunction and death, leading to cognitive dysfunction (Villa-Gonzalez et al., 2024; Huang et al., 2024). Continuous damage to the vascular system results in the destruction of the BBB, further promoting the inflammatory response and eventually leading to more severe cognitive dysfunction and even dementia (Tang et al., 2024; Nyul-Toth et al., 2024). Additionally, the complement system can also cause neuroinflammation. In the early stages of VCI, complement components (C1q and C3) facilitate phagocytosis and clearance of amyloid fibrils; however, in late stages, C5 activates the membrane attack complex to induce neuroinflammation and neuronal damage (Batista et al., 2024; Wen et al., 2024). In summary, the entire inflammatory process promotes astrogliosis, endothelial dysfunction, BBB disruption, and complement system disorders, leading to neuronal damage.

#### 2.1.3 Mitochondrial dysfunction

Mitochondria, known as the “powerhouse” organelles of the cell, provide 95% of cellular energy and play an important role many cell biological processes (Spinelli and Haigis, 2018; Hu et al., 2024). Ischemia and hypoxia reduces the synthesis of ATP in nerve cells, the Na^+^/K^+^ pump on the cell membrane is inactivated, and excitatory amino acid (mainly glutamate) levels increase, which activates the formation of mitochondrial permeability transition pore (mPTP). This leads to Ca^2 +^ overload and disrupts the mitochondrial membrane potential (Wang et al., 2023; Rahi and Kaundal, 2024). The open mPTP also allows other metabolites to enter the mitochondrial matrix space and uncouples the mitochondrial ETC from ATP synthase activity, leading to a reduction in ATP, cessation of oxidative phosphorylation, and finally, production of excessive ROS (Beg et al., 2024; He et al., 2022). ROS destroy the activities of complexes I to IV and impairs mitochondrial respiratory function, resulting in mitochondrial outer membrane damage. Cytochrome C and apoptosis-inducing factor in mitochondria are released into the cytoplasm and promote neuronal apoptosis (Okoye et al., 2023). Additionally, prolonged hypoxia, mitochondrial Ca^2+^ overload, and excessive ROS accumulation may completely inhibit or over-activate mitophagy, leading to mitochondrial homeostasis imbalance and further aggravating cerebral ischemic injury and cognitive impairment (Wang et al., 2024). Therefore, mitochondrial dysfunction is among the fundamental causes of nerve damage and cognitive impairment in VCI.

#### 2.1.4 Excitotoxicity

Excitotoxicity is one of the earliest discovered and widely recognized molecular mechanisms of injury after ischemic stroke (Burch et al., 2024). Due to disordered metabolism in the ischemic area in VCI, excessive release of the excitatory neurotransmitter glutamate in the synaptic cleft increases activation of the N-methyl-D-aspartic acid (NMDA), *α*-amino-3-hydroxy-5-methyl-4-isoxazole propionic acid, and kainic acid receptors, causing increased Ca^2+^ influx. The dramatic increase in intracellular Ca^2+^ activates neutral proteases, endonucleases, and phospholipases, resulting in the destruction of DNA and the neuronal cytoskeleton, simultaneously producing various free radicals and apoptotic bodies and eventually causing neuronal apoptosis (Rajeev et al., 2023; Deng et al., 2019). Moreover excitability toxicity and excessive NMDA receptor activation triggers the JNK/c-Jun/AP-1 signal transduction pathway, further expanding ischemic cell death (Nuzzo et al., 2019). Furthermore, NMDA activation stimulates NOS to produce a high concentration of NO, which further promotes neuronal damage (Wang et al., 2024; de Sousa Maciel et al., 2022). In summary, excitotoxicity is an important neurobiological phenomenon that plays a key role in various neurological diseases and injuries.

### 2.2 Neuropathological features of VCI

#### 2.2.1 Glial activation

Glial activation, particularly microglial activation, results in polarization towards proinflammatory M1 and anti-inflammatory M2 phenotypes (Ye et al., 2024; Lokesh et al., 2024). M1 microglia secrete a variety of proinflammatory cytokines, whereas M2 microglia secrete a series of anti-inflammatory cytokines that promote endocytosis and eventually reduce neuronal damage (Mo et al., 2022; Wang et al., 2024). Microglial cells in the brain of VCI patients multiply, especially in the white matter, and quickly activate, causing morphological changes (Zhao et al., 2024) that mainly promote the polarization of microglia cells to the proinflammatory M1 phenotype. Secreted proinflammatory factors aggravate brain tissue damage after ischemia (Pang et al., 2023). *In vitro* experiments showed that 2,3,5,6-tetramethylpyrazine had an anti-inflammatory effect, restraining the inactivation of the NF-κB signaling pathway to inhibit the polarization of M1 microglia and reducing the expression of inducible NO synthase and CD86 M1 markers and proinflammatory cytokines TNF-*α* and IL-6, thereby reducing lipopolysaccharide (LPS)-induced neuroinflammation (Chen et al., 2023). Another study showed that paeoniflorin inhibited activation of the NF-κB signaling pathway, thereby blocking the polarization of M1 microglia in the hippocampal CA1 region of VCI rats and reducing the expression of inflammatory mediators IL-1β, IL-6, TNF-α, and NO. Furthermore, paeoniflorin can upregulate the expression of anti-inflammatory factors IL-10 and TGF-β1 in M2 microglia, thereby reducing neuroinflammation in the hippocampal CA1 region (Luo et al., 2018). In conclusion, microglia can promote neuroinflammation in chronic cerebral ischemia by activating the NF-κB signaling pathway (Chen et al., 2024).

#### 2.2.2 Cell death

Programmed cell death is a regulatory mechanism mediated by signal transduction pathways. Abnormal regulation of programmed cell death is associated with neurodegenerative diseases and cancer (Yang et al., 2022). In CCH animal models, as ischemic time was prolonged, apoptosis of hippocampal neurons and Bax expression increased, while expression of the anti-apoptotic factor Bcl-2 decreased (Niu et al., 2021; Zhang et al., 2021). Expression of the autophagy markers P62 and LC3-II/LC3-I increased (Xu et al., 2023; Wang et al., 2023), whereas ferroptosis-related proteins such as solute carrier family 7 member 11 and GPX4 were downregulated (Fan et al., 2024; Lou et al., 2024). Additionally, expression of pyroptotic proteins caspase-3, nod-like receptor pyrin domain-containing protein 3 (NLRP3), GSDMD-N, caspase-1 p20 (its active form), and ASC were increased (Zhu et al., 2024; Zhang et al., 2023). Necroptosis plays an important role in hippocampal neuron loss and white matter damage after chronic cerebral ischemia (Nikseresht et al., 2019; Chen et al., 2018). Various cell death modes mediate VCI cell loss; however, necroptosis plays a particularly prominent role and is a potential target for regulating VCI neuron loss.

#### 2.2.3 BBB dysfunction

The BBB is an important physiological barrier between the CNS and peripheral blood circulation. It selectively transports nutrients, expels toxic substances and metabolites (Zhang et al., 2024; Zhou et al., 2024), and maintains the dynamic stability of the internal environment of the CNS (Shang et al., 2024; Bai and Ge, 2024).

The mechanism of BBB dysfunction in VCI is not fully understood; however, oxidative stress, excitotoxicity, and neuroinflammation promote dysregulation of EC apical tight junction proteins, leading to increased BBB permeability. This promotes the entry of cytokines, immunoglobulins, and self-secreted serum factors into the brain, resulting in collagen deposition around the corpus callosum. Subsequently, WMLs are formed, resulting in cognitive decline (Rajeev et al., 2022; Rajeev et al., 2022). Additionally, increased endothelial transendocytosis after CCH leads to BBB dysfunction (Shang et al., 2024). In cerebral ischemia, excitatory toxicity from increased Ca^2+^ concentration in the EC cytoplasm induces metabolic disorders, mitochondrial dysfunction, protease activity, and the activation of phospholipase and ROS generation. These common factors cause EC membrane injury and vascular cell death, damaging the integrity of the BBB (Shah et al., 2024; Zhao et al., 2022). Animal experiments have shown that CCH-induced cognitive impairment can be improved by alleviating neuroinflammation and protecting the BBB (Wang et al., 2023; Li et al., 2022).

#### 2.2.4 Neuronal demyelination and WMLs

WMLs are predominantly involved in brain structure changes, and the main pathological features are demyelination, gliosis, loss of nerve fibers and oligodendrocytes, and microglial activation (Lin et al., 2024; Xiao et al., 2023). WMLs are associated with a 73% increased risk of VCI (Hu et al., 2021). CCH induces oxidative stress, inflammation, oligodendrocyte apoptosis, and microglial activation, promotes demyelination, decreases nerve fiber microtubule-associated protein 2 (MAP2) levels, and increases myelin basic protein levels, leading to impaired cognitive function and further aggravating WMLs (Jiang et al., 2024; Guo et al., 2022). Therefore, extensive cerebral ischemia-induced WMLs are now considered the key drivers and most important pathological features of VCI and dementia and are an important cause of cognitive deficits.

Pathophysiological studies on cerebral ischemia suggest that glial cell activation, cell death pathways, BBB function, and therapeutic interventions for WMLs may inhibit the pathological progression of VCI.

## 3 Necroptosis

Necroptosis is a form of programmed cell death that differs from apoptosis and does not depend on caspase activity. Necroptosis is characterized by the activation of receptor-interacting protein kinase (RIPKs), followed by phosphorylation and activation of mixed lineage kinase domain-like protein (MLKL), resulting in plasma membrane rupture and the release of cellular contents. Eventually, the cytoplasm and nucleus disassemble, and the cell dies (Ai et al., 2024; Guo et al., 2024).

### 3.1 Molecular mechanisms and signaling pathways of necroptosis

Necroptosis relies mainly on death receptor activation, including TNF receptor 1 (TNFR1), Fas/CD95, and TNF-related apoptosis-inducing ligand (TRAIL) receptors (Wu et al., 2024; Zhou et al., 2024).

#### 3.1.1 Classical pathway

**Figure 2:**
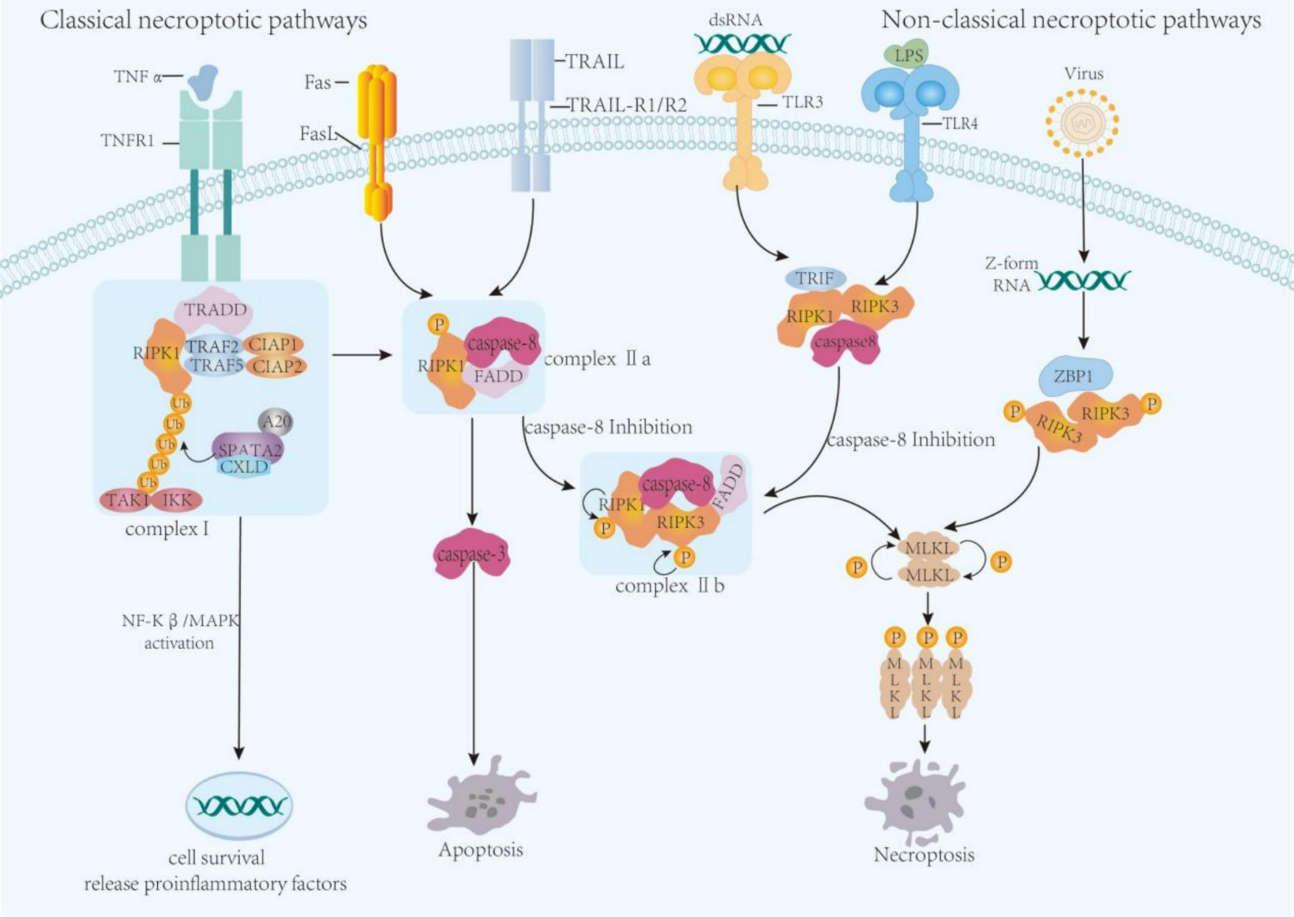
Necroptotic pathways. In the classical necroptosis pathway, TNF-*α* binds to its receptor TNFR1 and then to TRADD, RIPK1, TRAF family proteins, cIAP1/2, and LUBAC to form complex I, which is critical for cell survival. In most cases, TRADD in complex I acts as an adaptor molecule that, once activated, recruits RIPK1 to TNFR1, cIAP1/2, and TRAF2/5 to promote complex I stabilization. TGF-*β*-activated kinase 1 is then activated to recruit IKK complexes. This leads to the activation of the NF-κB pathway, which promotes the production of inflammatory factors and cell survival. CYLD is recruited to complex I through its adaptor protein spermatogenesis-associated 2, which in turn binds to LUBAC. The proximity of CYLD to its substrate depends on the ubiquitin-binding protein A20. Once CYLD mediates the deubiquitination of RIPK1, the stability of complex I decreases. RIPK1 dissociates from complex I on the plasma membrane and recruits Fas-associated death domain protein (FADD), which then binds to pro-caspase-8 to form complex IIa. In complex IIa, the caspase-8 complex activates the caspase signaling pathway to induce apoptosis, thereby triggering cell death. When caspase-8 is inhibited, RIPK1 is autophosphorylated and binds to RIPK3 via the RIPK homotypic interaction motif to form complex IIb, and RIPK3 phosphorylation promotes MLKL phosphorylation. p-MLKL oligomerizes and translocates to the cell membrane, leading to the disruption of cell and organelle membranes and eventually to necrosis. The non-classical necroptotic pathway is complex and diverse. Double-stranded RNA molecules recognize and bind TLR3 and LPS recognizes and binds TLR4 and recruits phosphorylated RIPK1 and RIPK3 by binding TRIF, leading to programmed necrosis. ZBP1 recognizes and binds to Z-form RNA molecules released by virus-released nucleic acids in the cytoplasm, and simultaneously activates and phosphorylates RIPK3, which acquires kinase activity. Activated RIPK3 phosphorylates MLKL and induces necroptosis.

In the classical necroptotic pathway, TNF-*α* binds to its receptor TNFR1 and then combines with TNF receptor-associated death domain protein (TRADD), RIPK1, TNF receptor-associated factor family proteins (TRAFs), and inhibitor of apoptosis protein cIAP1/2 and linear ubiquitin chain assembly complex (LUBAC) to form complex I (Wu et al., 2024; Wendlocha et al., 2024). In response to deubiquitinating enzymes such as cylindromatosis (CYLD), RIPK1 dissociates from complex I and recruits Fas-associated death domain protein (FADD), which then binds to pro-caspase-8 to generate complex IIa. When caspase-8 is inhibited, RIPK1 is autophosphorylated at Ser166 and binds to RIPK3 via the RIPK homotypic interaction motif to form complex IIb, and RIP3 is phosphorylated at Ser227 to promote MLKL phosphorylation. Phosphorylated MLKL (p-MLKL) oligomerizes and translocates to the cell membrane, leading to the disruption of cell and organelle membranes and eventually to necrosis (Ranjan and Pathak, 2024; Tran et al., 2024) (Figure 2).

#### 3.1.2 Non-classical pathway

Necroptosis involves several non-classical pathways. Although the activation pathways are not identical, RIPK and MLKL are activated. For example, Z-DNA binding protein 1(ZBP1) activates RIPK3-dependent cell death upon binding to Z-form nucleic acids, including Z-RNA produced by certain viral or endogenous retroviral elements (Yang et al., 2023; Liu et al., 2023). Toll-like receptor 3 (TLR3) recognizes and binds to double-stranded RNA molecules in the cytoplasm and recruits and phosphorylates RIPK1 and RIPK3 via the bound toll-like receptor linker molecule 1 (TRIF). TLR4 is a membrane receptor that combines with LPS signaling molecules and relies on TRIF recruitment of RIPK1 and RIPK3 (Muendlein et al., 2022; Chen et al., 2022). Type I/II interferon can activate double-stranded RNA-dependent kinase, which binds RIPK1 and RIPK3 (Rosenberg and Sibley, 2021). TRAIL can convert apoptosis into an RIPK1-dependent necrosis pathway in an acidic environment (Park et al., 2020). TNFR lacking the death domain can initiate necrosis via the RIPK1-FADD-caspase-8 complex (Abd El-Aal et al., 2022; Pham et al., 2019). In some cases, MLKL can be directly activated independently of RIPK3 activation.

## 4 Necroptosis in VCI

Clinical research on VCI is usually based on animal models of cerebral hypoperfusion and ischemia. In 2005, Degterev et al. (2005) first demonstrated the existence of necroptosis via a mechanism different from that of apoptosis in a mouse model of ischemic brain injury. Subsequently, an increasing number of studies have shown that necroptosis plays an important role in the pathogenesis of cerebral ischemic lesions (Deng et al., 2019; Fan et al., 2024). Increased expression of necroptosis markers such as TNF-*α*, RIPK3, and MLKL is observed in animal models (Chevin et al., 2022). Increased expression of RIPK1 and RIPK3 has been observed in oxygen and glucose deprivation (OGD)-induced cell injury models *in vitro* (Ni et al., 2018; Zhu et al., 2021). Therefore, necroptosis can be used as a new therapeutic target in VCI. Below, we review necroptosis in VCI pathological changes.

### 4.1 Necroptosis and neuroinflammation

The essence of classical necroptotic pathway is that TNF-α triggers a “programmed” proinflammatory form of cell necrosis by binding to its receptor TNFRI (He et al., 2024). During VCI cerebral ischemia, MLKL is phosphorylated and translocates to the plasma membrane, leading to an influx of Ca^2+^ and Na^+^ ions, which immediately opens the mPTP. Inflammatory cell damage-associated molecular patterns such as mitochondrial DNA, high mobility group box1 (HMGB1), and IL-1 are released (Prasad Panda et al., 2023). HMGB1is released from the nucleus to the cytoplasm and binds directly to TLR4 to activates NF-κB, thereby promoting the production of various proinflammatory mediators (Albaqami et al., 2024). HMGB1 inhibition in the brain tissue of cerebral ischemia mice models and *in vitro* studies increases TBK1 and IFNβ encephalitis markers and decreases NF-κB (Saeedan et al., 2023; Li et al., 2023). Additionally, NLRP3 or caspase-8 can activate inflammasome formation in response to RIPK3. Activation of the TLR4 signaling pathway promotes NF-κB to activate IL-1β, IL-18, and other precursors (Jiang et al., 2024; Zhang et al., 2024). Researchers have focused on neuronal necroptosis in VCI. Targeting RIPK1/RIPK3 and MLKL may help overcome therapeutic barriers in the treatment of neuroinflammatory and neurodegenerative diseases and in preventing neuronal necroptosis.

### 4.2 Necroptosis and neuronal cell death

The expression of necroptosis kinases, including MLKL/p-MLKL, RIPK3/p-RIPK3, and RIPK1/p-RIPK1, was significantly increased in the brain tissue of an OGD-induced neuronal injury model and the brain tissue of cerebral ischemia animal models (Fan et al., 2024; Yan et al., 2022). Moreover, the levels of neuronal necrotizing factors and colocalization of NeuN and MLKL were also increased in middle cerebral artery occlusion (MCAO) mice (Huang et al., 2023), indicating that necroptosis is involved in neuronal death in cerebral ischemia. Vieira et al. (2014) explored the mechanism of OGD-induced necroptosis in hippocampal neurons *in vitro* and observed that the upregulation of RIPK1 and RIPK3 protein levels induced by ischemic injury was positively correlated with the death of hippocampal neurons. RIP1 kinase blockade showed a significant neuroprotective effect, resulting in a 66% survival rate in the “ischemic” group, compared to an 83% survival rate in animals given the RIPK inhibitor necrostatin (Mitroshina et al., 2022). Necroptosis may play an important role in cell death and neuronal damage caused by cerebral ischemia.

### 4.3 Necroptosis and BBB

The BBB is a unique microvascular system, and ECs of the brain microvascular system constitute a key component of the BBB (Sweeney et al., 2019; Bellut et al., 2021). As mentioned previously, cerebral ischemic injury leads to disruption of BBB function and a significant increase in its permeability. Levels of the necroptosis biomarkers pRIPK1, pRIPK3, and p-MLKL increase in brain tissues, and they colocalize with the cerebral microvascular EC marker CD31 (Li et al., 2023; Wang et al., 2021). After administration of RIPK1 inhibitor, necrostatin 1, endothelial necroptosis and BBB leakage were significantly decreased (Chen et al., 2019; Chen et al., 2019). Furthermore, in a mouse *RIPK1* and *MLKL* double knockout stroke model, the permeability of the BBB is significantly reduced (Lule et al., 2021). This indicates that brain ischemic injury activates programmed cell death of vascular ECs, leading to dysfunction of the BBB.

### 4.4 Necroptosis and demyelination

Oligodendrocytes form myelin to accelerate axon impulse conduction. In OGD-induced cells and permanent MCAO models, the number of neurons and astrocytes expressing RIP1K, RIP3K, and RIP1K-RIP3K complexes is increased, and MAP2 and GFAP levels are decreased. RIP1K knockdown or necrosis inhibition-1 (Nec) treatment reduces the necroptosis of neurons and astrocytes, thereby inducing demyelination (Ni et al., 2018). Demyelination has been observed in other neuropathies such as multiple sclerosis (MS) and spinal cord injury (SCI). TNFα levels are increased in the serum, brain, and cerebrospinal fluid of patients with MS, and RIPK1 is activated in mature oligodendrocytes and neurons in animal models and patients with MS (Picon et al., 2021; Zelic et al., 2021). RIPK1 and the oligodendrocyte marker IBA1 are colocalized in brain tissue (Ma et al., 2022). Administration of RIPK1 inhibitors protected mature oligodendrocytes from death and alleviated symptoms of nerve injury in a mouse model of MS, suggesting that inhibition of RIPK1 activity inhibits demyelination (Yoshikawa et al., 2018; Fan et al., 2019). RIPK3/MLKL-mediated necroptosis causes oligodendrocyte death and demyelination in SCI (Fiani et al., 2021; Song et al., 2024). Therefore, necroptosis may play an important role in nerve demyelination in VCI.

Few studies have investigated necroptosis in VCI. However, an increasing number of researchers have focused on the importance of necroptosis in cranial nerve injury. Therefore, targeting necroptosis pathway proteins for VCI therapy is feasible, and several necroptosis pathway inhibitors have been developed for experimental treatments.

## 5 Necroptosis is the target of VCI treatment

Existing small-molecule inhibitors targeting key proteins in the necroptotic pathway have shown promising therapeutic effects. In recent years, the application of nanotechnology with functional materials and biomedical science fusion technology to induce or inhibit necroptosis has provided great opportunities for the treatment of diseases and may be a potential therapeutic strategy.

### 5.1 Necroptosis inhibitors

RIPK3, RIPK1, and MLKL play important roles in neuronal necroptosis in VCI. Therefore, targeted inhibition of these kinases can improve neuronal necroptosis and thus improve cognitive impairment in VCI.

#### 5.1.1 RIPK1 inhibitors

RIPK1 has a unique hydrophobic pocket that modulates its kinase activity through structural changes. All RIPK1 inhibitors reported to date bind to this pocket (Xie et al., 2013). Nec-1 was first discovered by screening necroptotic inhibitors (Chevin et al., 2022). Nec-1 significantly reduces ischemia-induced RIPK1 and increases the number of neurons in a rat MCAO cerebral ischemia model, thus showing a protective effect on brain injury (Deng et al., 2019). In an LPS-induced neuritis mouse model, Necs can inhibit microglia activation by inhibiting RIPK1 phosphorylation, thereby inhibiting neuroinflammation (Kim et al., 2023). GSK481, GSK772, GSK963, and GSK547 are also RIPK1 inhibitors (Mifflin et al., 2020). RIPK1 inhibitors such as SAR443820, SIR2446M, GFH312 have been tested in phase I clinical trials in healthy subjects, It has a good safety, pharmacokinetic and pharmacodynamic profile (Hincelin-Mery et al., 2024; Sun et al., 2024; Lickliter et al., 2023). RIPK1 inhibitors showed various neural inflammatory disease treatment effects but did not specifically inhibit necroptosis because they can also inhibit apoptosis.

#### 5.1.2 RIPK3 inhibitors

Unlike RIPK1, RIPK3 does not affect apoptosis, and targeted inhibition of RIPK3 more specifically controls necroptosis (Prasad Panda et al., 2023). Therefore, specific RIPK3 inhibitors are important for necroptosis-related drug research and development. Classical RIPK3 small-molecule inhibitors include GSK840, GSK84, GSK872, and GW39B (Sun et al., 2024; Xu et al., 2023). GSK872 administration decreased RIPK3, p-JNK, and IL-6 expression and neuronal death and improved neurobehavior in rats (Hu et al., 2020). Moreover, GSK872 inhibited neuronal necroptosis in intracerebral hemorrhage mice through the death domain-associated protein signaling pathway (Bai et al., 2024). Many RIPK3 inhibitors, such as AZD5423, Compound-42, and Zharp-99, have been developed to inhibit necrotizing apoptosis in mouse inflammatory models and acute kidney injury, thereby alleviating kidney injury and systemic inflammation (He et al., 2023; Xu et al., 2022). Therefore, RIPK3 inhibitors could also have a neuroprotective effect in VCI.

#### 5.1.3 MLKL inhibitors

MLKL, which comprises a four-helix bundle (4HB) and pseudokinase (psK) domains, acts as an executor of necroptosis and is an important drug target, and many inhibitors have been tested in previous studies. These include covalent inhibitors, ATP competitive inhibitors, and noncovalent inhibitors (Tang and Zhuang, 2024; Martinez-Osorio et al., 2023). As irreversible covalent inhibitors, necrosulfonamide and compound TC13172 both bind to Cys86 of human MLKL to protect cells from necroptosis; however, they do not affect necroptosis in mouse cells (Ji et al., 2021). GW806742X was the first molecule discovered that protects the cell from necroptosis by competing with ATP binding at the psK domain of MLKL (Pierotti et al., 2020). Another noncovalent inhibitor binds to the 4HB domain of MLKL, thereby inhibiting its action (Cui et al., 2022). However, the current molecular regulators of MLKL are in the early drug development stage and still have some limitations, so they are mainly applied in research.

### 5.2 Nanotechnology targets necroptosis

Nanotechnology can overcome the traditional drug delivery problem and has attracted much attention in the diagnosis and treatment of diseases (Zhang et al., 2019; Zang et al., 2022). The surface of nanoparticles can carry various therapeutic small-molecule drugs, proteins, peptides and proteins, small interfering RNA, microRNA, and DNA, which can recognize and bind to target cells (Zhang et al., 2019). The main limitation of VCI-targeted therapy is that drugs cannot cross the BBB easily, limiting their delivery to the target site. Nanotechnology can solve these problems by improving the pharmacokinetics of drugs and obtaining better neurovascular access (Amani et al., 2019). Few studies have been conducted on nanotechnology targeting necroptosis for treating VCI. However, loaded nanoparticles have a significant neuroprotective effect in a cerebral ischemia model, and targeting necroptosis in tumors has a significant effect.

The oxidative stress products ROS and MDA increase during cerebral ischemia, and their inhibition can improve brain tissue damage. Baicalin loaded onto cyclodextrin and polyethylene glycol–polylactic acid-co-glycolic acid (PEG-PLGA) to form polyethylene PEG-PLGA nanoparticles (PEG-PLGA RNP) significantly reduced the levels of MDA and ROS in the brain tissue of MCAO rats (Li et al., 2022). ROS-responsive chitosan-bilirubin nanoparticles loaded with Statin (ChiBil-Statin) reduced ROS production in an OGD-induced cell model by approximately 5.6-fold (Raveena et al., 2024). Inhibition of HMGB1 can reduce brain damage caused by neuroinflammation. 8β-glycyrrhetinic acid is a potent intracellular HMGB1 inhibitor. ROS-responsive polymer-drug conjugate nanoparticles (diglycolic acid) designed to prevent the translocation of HMGB1 inhibited the polarization of microglia to the M1 phenotype both *in vitro* and *in vivo* (Jin et al., 2023). Direct administration of neutrophil-mediated nanoparticles to the cerebral ischemic area can significantly reduce the infarct volume in MCAO mice, thereby improving cognitive impairment (Zhang et al., 2017). In conclusion, nanotechnology can reduce neuroinflammation and oxidative stress in cerebral ischemia and plays a key role in neuroprotection.

Targeting cancer cells with nanoparticles to induce necroptosis is widely used in cancer therapy. Liang et al. (2024) developed a MUC1 aptamer-targeting nanocomposite (MUC1@Chi-Ag@CPB@SHK, MUC1@ACS) for delivering shikonin and chitosan silver nanoparticles. The accumulation of MUC1@ACS nanoparticles at the tumor site increased by 6.02 times. Upregulation of RIPK3, p-RIPK3, and tetramer MLKL expression synergistically induces tumor cell necroptosis. Iron-palladium nanozyme and shikonin-encapsulated functional lipid nanoparticles increase ROS production in tumor cells and promote programmed cell death, thereby inhibiting tumor cell growth *in vitro* (Xie et al., 2024).

Nanotechnology targeting necroptosis in the treatment of VCI has not been reported; however, it has shown good neuroprotective effects against cerebral ischemic injury and a significant tumor-inhibitory effect. Therefore, we reasoned that nanotechnology targeting necroptosis in VCI treatment should be neuroprotective.

## 6 Conclusion and future prospects

This review elaborates on the potential pathophysiological mechanisms of VCI. Any one of these pathological mechanisms can trigger a vicious cycle of accelerated brain damage. However, these mechanisms have not been fully elucidated.

Necroptosis is mainly induced by extracellular factors (TNFα) and participates in pathophysiological processes in various diseases, such as neurodegenerative diseases, cancer, and autoimmune diseases. The intersection of necroptosis and apoptosis regulation increases the complexity of related pathways, such as caspase-8, FADD, and RIPK1, which are programmed in the cell apoptosis and necroptosis pathways in complex relationships. Additionally, necroptosis varies among tissues and is closely associated with VCI occurrence. Studies have shown that involvement of neuroinflammation and necroptosis in the hippocampus during vascular dementia in post-mortem brain examinations, However, research on necroptosis in VCI is limited, and the degree of its effect remains unclear.

Several necroptosis inhibitors targeting necroptosis pathway proteins have been developed as experimental treatments. Blockade of necroptosis by RIPK1-, RIPK3-, or MLKL-targeting therapy can effectively reduce oxidative stress, neuroinflammation, and neuronal death caused by ischemic brain injuries. Nanotechnology has also demonstrated good neuroprotective effects against cerebral ischemic injury. Therefore, nanotechnology targeting necrotizing ptosis may be a potential neuroprotective strategy to alleviate VCI brain damage. However, the extent of necroptosis compared to other pathways of death remains to be clarified. Second, although new necroptosis inhibitors with neuroprotective potential have been studied in phase 1 clinical trials, research is still in its early stages and the cytotoxicity of these inhibitors remains to be elucidated. Finally, necroptosis in the ischemic core and peri-infarct areas of the brain in stroke patients is most convincing; However, it is limited by the small sample size.

In conclusion, necroptosis is involved in the formation and progression of VCI, and interventions targeting necroptosis may effectively control neural injury after cerebral ischemia. Therefore, necroptosis inhibition may be a new target for the prevention and treatment of ischemic stroke.
